# Public health communication: Attitudes, experiences, and lessons learned from users of a COVID-19 digital triage tool for children

**DOI:** 10.3389/fpubh.2022.901125

**Published:** 2022-08-01

**Authors:** Janet Michel, Julia Rehsmann, Annette Mettler, Carl Starvaggi, Nicola Travaglini, Christoph Aebi, Kristina Keitel, Thomas C. Sauter

**Affiliations:** ^1^Department of Emergency Medicine, Inselspital, Bern University Hospital, University of Bern, Bern, Switzerland; ^2^Emergency Telemedicine, University of Bern, Bern, Switzerland; ^3^School of Health Professions, Bern University of Applied Sciences, Bern, Switzerland; ^4^Pediatric Emergency Medicine, Department of Pediatrics, Inselspital, Bern University Hospital, University of Bern, Bern, Switzerland; ^5^Department of Pediatrics, Inselspital, Bern University Hospital, University of Bern, Bern, Switzerland

**Keywords:** children, COVID-19, childcare, digital triage, public health communication, quarantine, testing

## Abstract

**Background:**

The pandemic has made public health communication even more daunting because acceptance and implementation of official guidelines and recommendations hinge on this. The situation becomes even more precarious when children are involved. Our child-specific COVID-19 online forward triage tool (OFTT) revealed some of the public health communication challenges. We aimed to explore attitudes, experiences, and challenges faced by OFTT users and their families, in regard to public health recommendations.

**Methods:**

We selected key informants (*n* = 20) from a population of parents, teachers, guardians, as well as doctors who had used the child-specific COVID-19 OFTT and had consented to a further study. Videos rather than face-face interviews were held. Convenience and quota sampling were performed to include a variety of key informants. Interviews were recorded, transcribed verbatim, and analyzed for themes.

**Results:**

Several themes emerged, namely; (1) definition and expectations of high-risk persons, (2) quarantine instructions and challenges, (3) blurred division of responsibility between authorities and parents, (4) a novel condition and the evolution of knowledge, (5) definition and implications of socioeconomic status, (6) new normal and societal divisions, and (7) the interconnectedness of these factors-systems thinking.

**Conclusion:**

As the virus is evolving and circumstances are changing rapidly, the communication of public health to the different interest groups becomes, both an art and science, even more so when using a new technological communication channel: an OFTT. A myriad of interconnected factors seems to influence attitudes toward public health recommendations, which calls for systems thinking in public health communication.

## Background

In February 2020, the first cases of COVID-19 were confirmed in Switzerland, forcing the country into a lockdown, which included a temporary closure of day care and primary schools. Guided by the directive “Bleiben Sie zuhause/Stay at home,” people were asked to work from home, avoid public transport, as well as limit social contacts (1–5). With many parents and children suddenly at home and an unknown virus lurking, public health communication became critical. The way public health messages are communicated has a bearing on the acceptance and implementation of official guidelines, and recommendations, as well as general societal reassurance and family wellbeing (4). Uncertainty about the virus and about recommendations, disruption, and postponement of events, school, and social ones seem to elevate stress levels and impact mental health in children (5, 6). Consequently, mental health problems grew during lockdowns, especially among children and adolescents (6, 7).

Public health recommendations and measures to prevent infections had to be adapted to the evolving knowledge and virus (8–10). Health authorities scheduled regular communication briefings to inform and reassure the public. The way information is conveyed shapes public trust both negatively and positively. According to the literature, adherence and attitudes to public health recommendations in Switzerland differ along cultural, geographical, and socioeconomic lines, highlighting the importance of targeted communication (11).

Switzerland is governed by a federal system; the majority of public health and education policies fall under the responsibility of the 26 cantons with a strong focus on individual responsibility. During some phases of the pandemic, decision-making was centralized through a pandemic emergency law, and key public health recommendations were issued by the federal office of public health (FOPH), including a pediatric COVID-19 testing strategy. The Swiss FOPH held regular media releases throughout the pandemic (12). The primary objective of communication in public health crises is to influence behavior so as to reduce the duration and impact of the crises, e.g., the COVID-19 pandemic (13). Public health communication depends on trust. The message needs to be clear on the urgency and practical behavior recommended, specifically packaged to meet the varying needs of different groups in society (14).

Telehealth became an essential component of the Swiss healthcare system, as a result of the pandemic (15, 16). The introduction of online forward triage tools (OFTTs) for healthcare and public health communication has created a new and potentially scalable, public health communication channel. This channel has the potential to reach large numbers of people, irrespective of the time of tool use and location of the user. OFTTs have been developed to communicate public health recommendations regarding testing, isolation/quarantine, as well as advice regarding accessing healthcare services, and school or day-care attendance. Unique attributes of a child-specific OFTT are the different levels and recipients of the recommendation, ranging from the affected patients, the children, their families, and caregivers to a population-wide audience.

The purpose of this study was to explore attitudes, experiences, and challenges faced by Swiss OFTT users in regard to public health recommendations given by a child-specific COVID-19 OFTT (pandemic context).

## Methods

### Context and intervention

The Department of Emergency Telemedicine of the University of Bern together with Paediatric Emergency Medicine, the Department of Paediatrics, Inselspital, Bern University Hospital, University of Bern, Switzerland, and the Department of Paediatrics, Inselspital, Bern University Hospital, University of Bern, Switzerland developed a child-specific COVID-19 OFTT, www.coronabambini.ch. The goal of the OFTT was to provide parents and guardians with public health recommendations with regards to testing, isolation, quarantine, school or day-care attendance, and when to seek healthcare. Details on the development and structure of the OFFT including quantitative data on its usage have been published elsewhere (17).

### Study design

An exploratory qualitative study design was utilized. The overarching aim of this study was to assess the utility of the child-specific COVID-19 OFTT, www.coronabambini.ch, as well as elicit recommendations to improve future OFTTs. This study aimed to explore the attitudes, experiences, and challenges of Swiss OFTT users and their families, with regard to the communicated public health recommendations of www.coronabambini.ch. We explored the following questions:

Central question

- What was your lived experience of COVID-19 with respect to testing, quarantine, and public health communications?

Sub-questions

- What made you follow or disregard the recommendations given by the OFTT?
- What is your understanding of the following terms: socioeconomic status, infectiousness of children, and high-risk person?
- How did these experiences (testing, quarantine, and public health communications) influence your decisions to follow or not follow the OFTT recommendations?

### Sampling and sample size

We adopted a purposeful and quota sampling approach, with a total of key informants (*n* = 20) selected from a population of parents, teachers, and guardians, including persons with dual roles as a parent and healthcare/school professional, who had used www.coronabambini.ch, and consented to the study (Table 1). In qualitative research, saturation guides sample size, and we aimed for both rich narratives and thematic saturation (18, 19). This was reached by the 12th key informant.

**Table 1:** Key informants.

**Guardian roles**	**Females**	**Males**	**Total**
Mother role	10	–	10
Mother plus health care professional, teacher	2	–	2
father And medical professional	–	2	2
Father role		4	4
Father, Medical professional and or school authority		2	2
Total	**12**	**8**	**20**

### Data collection

Videos rather than face-face interviews were held in view of the public measures in place at the time of the interviews. An interview guide was used and adapted iteratively (Appendix). Two researchers were present in each session and filled the questions in turn. The interviews lasted for 45–55 min. Interviews were recorded and transcribed verbatim.

### Data analysis

An inductive and deductive approach to data analysis was utilized. Transcribed interviews were coded thematically (guided by a framework derived from a previous OFTT evaluation under review) (20) while remaining open to new themes. Data management and analysis were performed with the aid of MAXQDA2020 (VERBI Software, Berlin). Narratives on the patient's lived experience of testing, quarantine, and public health communication were elicited.

### Measure to ensure the trustworthiness of the data

Data collection and analysis were performed iteratively, continuously adapting the interview guide to ensure dependability. The two qualitative researchers debriefed at the end of each interview and kept reflexive journals. To ensure transferability, a thick description of participants, context, and data collection process has been outlined.

### Ethics approval

The evaluation of OFTT use was deemed a quality evaluation study by the ethics committee of the Canton of Bern, Switzerland (req-2020-01179). The need for a full ethical review was waived on 21 October 2020.

## Results

The following themes emerged: (1) definition and expectations of high-risk persons, (2) quarantine instructions and challenges, (3) blurred division of responsibility between authorities and parents, (4) a novel condition and the evolution of knowledge, (5) definition and implications of socioeconomic status, and (6) new normal and societal divisions (Table 2).

**Table 2:** Summary of emergent themes.

**Theme**	**Category**	**Unit meaning**
High risk person definition and expectations	Those aged above 65 Those with chronic illnesses	- The elderly usually grandparents - Some young parent with children
Quarantine instructions and challenge	Inactivity of kids Living space	- More screen time for many kids, overweight - Big apartments and houses and gardens made it easier
Blurred responsibility between authorities and parents	Federal office Canton authorities School authorities	- Different messages at times, who to follow?
A novel condition and the evolution of knowledge	Evolving virus Knowledge gap	- Symptoms, transmission and complications not known - everchanging recommendations
Socio economic status definition and implications	Term not known Sensitive info	- What is that, not understood by some - Some felt uncomfortable divulging status (privacy)
New normal and societal divisions	Fear Inter and intrafamilial conflict Old vs. young	- A runny nose - Protect grandparents

### Theme 1 High-risk person definition and expectations

Several public health recommendations were formulated to protect individuals at high risk of severe infection from COVID-19. Users were challenged in understanding this definition and its practical implications. Who is a person at high risk? What was expected from those that fell into this category? How did this category shift over time? Most key informants cited age, 65 years and older, and those with chronic conditions such as hypertension and diabetes, as the intended high-risk groups. Others were more than irritated by the high-risk label as revealed below:

“*I heard from BAG that auto-immune disease is a high-risk factor and was scared to death. I feared that both my husband and I (who have the same condition) were in mortal danger. I was constantly in fear that the kids would bring the virus home. Eventually auto-immune treatment was adapted for COVID patients-giving us the necessary encouragement. I was angry that – even though many factors were considered high risk-nothing was done to ensure the safety of these people. The only focus was on old people and not on me or us?”* [Key Informant 17]

“*My husband and I are both slightly overweight - so-called “high risk”. Should we stop caring for our kids during the pandemic?*” [Key Informant 15]

“*Day-care (KITA) workers do not disclose their status if they are at high risk. So, we took the kid out of KITA during the first wave – and that was our way of coping with the fear*.” [Key Informant 14]

“*I do think that the state has acted reasonably. I feel perhaps more could have been done, especially for people in high-risk situations*.” [Key Informant 11]

### The family construct, grandparents, meaning, and roles

Intergenerational responsibility is still embedded in society. Many families in Switzerland rely on grandparent support in childcare. The public health recommendations had substantial implications on families. It was a difficult task to balance family constructs with protecting family members in a situation of evolving information. There is a link between the high-risk definition as discussed earlier and the role of grandparents, most of whom automatically fell into the over 65 age group.With social distancing and closure of many public spaces, the role of the family became even more pronounced. The advent of the COVID-19 vaccines brought hope as grandparents were vaccinated and could resume their roles in the family.

“*My parents have not regularly, but still relatively often looked after the children, which we actually then stopped with the outbreak of the pandemic. My parents are both over 70, and the whole information situation made it difficult, because there was a very long discussion about the role of children in spreading the disease. We certainly were rather cautious to protect our parents. And only then, over time, did we occasionally allow them to look after the children wearing masks. And now they have both been vaccinated twice.”* [Key Informant 14]

“*People were pretty careful not to associate with people whose children didn't go to school together. That was a big issue. Also, we kept seeing the grandparents through the windows, but we didn't visit them anymore*.” [Key Informant 1]

“*Old people's homes, the grandparents and elderly, the situation and settings need revisiting-the human being is a social being-isolation dehumanizes people. There are issues of self-protection vs protection of others and individual autonomy vs communal responsibility.”* [Key Informant 11]

### Theme 2 Quarantine instructions and challenges

How does a family's housing situation affect people's experience of isolating, homeschooling, and working from home? Challenges experienced with quarantine recommendations varied widely ranging from space challenges and home office to quarantining with small children to health issues and homeschooling as revealed below:

“*I have seen many children's weight curves go up. The children moved less and were often at home. And also, the school situation I think was difficult for a lot of kids. For kids with latent hyperactivity, home-schooling didn't go well at all. I had several patients who were not doing well at all*.” [Key Informant 20]

“*I am not a teacher, nor do I want to be one. Now I have to teach my 3 children, attending different grades, algebra to history? I am overwhelmed*.” [Key Informant 1]

Most key informants revealed how they let their guard down as the pandemic lingered, a term known as pandemic fatigue. Below is what was said by some participants:

“*First quarantines are viewed as adventure but subsequent ones are viewed negatively and people try to avoid them.”* [Key Informant 10]

“*In the beginning it was bad. I also wore a mask when the child was sick because I didn't know what to do... but we are in the fortunate position that we have two floors. That's when we said the sick child is upstairs and in the room with me and my husband is in the basement. We also tried not to have dinner together. I have to say, that was at the very beginning, during the first lockdown. During the second lockdown, it was wintertime and you had a cold and you had a cough. Then it was no longer taken so, I can say seriously [laughs]? That maybe a wrong word. But the first time there was really just the uncertainty and we tried hard, with masks, with separate bedrooms. No hugs, no kisses.”* [Key Informant 1]

### Theme 3 Blurred responsibility between authorities and parents

Informants revealed challenges in following the recommendations of the OFTT based on national testing recommendations, citing blurred responsibility between different authorities and between parents and authorities. Who is responsible for the wellbeing of children, the parents, or the authorities? If the authorities have a say, which authority directives should parents follow? The complexities of this issue are revealed below:

“*Honestly. The school authorities say follow the canton's lead. The canton says they basically recommend this and that, and then each school develops its own concept. What do I do then as a mother? You are faced with a situation where everyone says it's the other person's fault.”* [Key Informant 15]

“*One cannot take kids out of school; it is not allowed. As parent you feel helpless At least they could offer online schools as an option if parents want to keep their kids safe*.” [Key Informant 2]

It is of interest that the problem is not only the communication gap but rather perceived shame or secrecy around COVID-19 cases (21), as revealed below:

“*Schools did not share the information of how many kids tested positive in their school freely. There was perhaps a sense of shame and guilt?”* [Key Informant 2]

### Theme 4 The evolution of knowledge about the virus

Evolving understanding of the pandemic and related uncertainty brought about fear as revealed below:

“*Then it was said again and again, no, they [children] are not a risk, etc., then it was said that they were. It is a very difficult situation. The logistics and everything, the work, there's so much involved that it was sometimes really hard to decide what to do.”* [Key Informant 19]

### Theme 5 Implications of socioeconomic status and challenges around its definition

Socioeconomic status seemed to play a role in the pandemic, though participants had less understanding of the term and concept.

“*Socio-economic status-what is that? What does that word mean?”* [Key Informant 7]*-*

“*That question disturbed me. I felt my privacy invaded and so did not answer the question. Explain why you want to know this and guarantee that the data will not be shared with third parties.”* [Key Informant 11]

Who can afford to test, isolate, and wait for results or quarantine at home in case of a positive test result? Gardens became a socioeconomic status symbol that was well-appreciated during the pandemic as revealed below:

“*We live in a house with a garden, luckily [laughs], especially during the lockdown that was great.”* [Key Informant 14]

“*We were privileged and fortunate that we were both able to work from home for the most part, and that we could share the child care, and that our children were not so young anymore*.” [Key Informant 11]

### Theme 6 New normal and societal divisions

Existing societal divisions became widened by the pandemic and mistrust fuelled by fear grew as revealed below:

“*In small villages, rumours abound. One father came to me and said that the neighbours went away for holiday and brought the infection and now I have the disease. My kid got it from their kid upon returning from holidays and brought it home to me. Needless to say that their father had not tested their child to confirm who brought the virus home. When I asked him why he did not test the child, he said that he was scared of exposing the child to the painful and potentially dangerous test but was anyway convinced of where it came from.”* [Key Informant 11]

“*What was known as a light flu before 2020, became a scary thing after 2020, and symptoms like a slight sore throat or runny nose almost meant isolation -literally and physically.”* [Key Informant 11]

## Discussion

**Figure 1:**
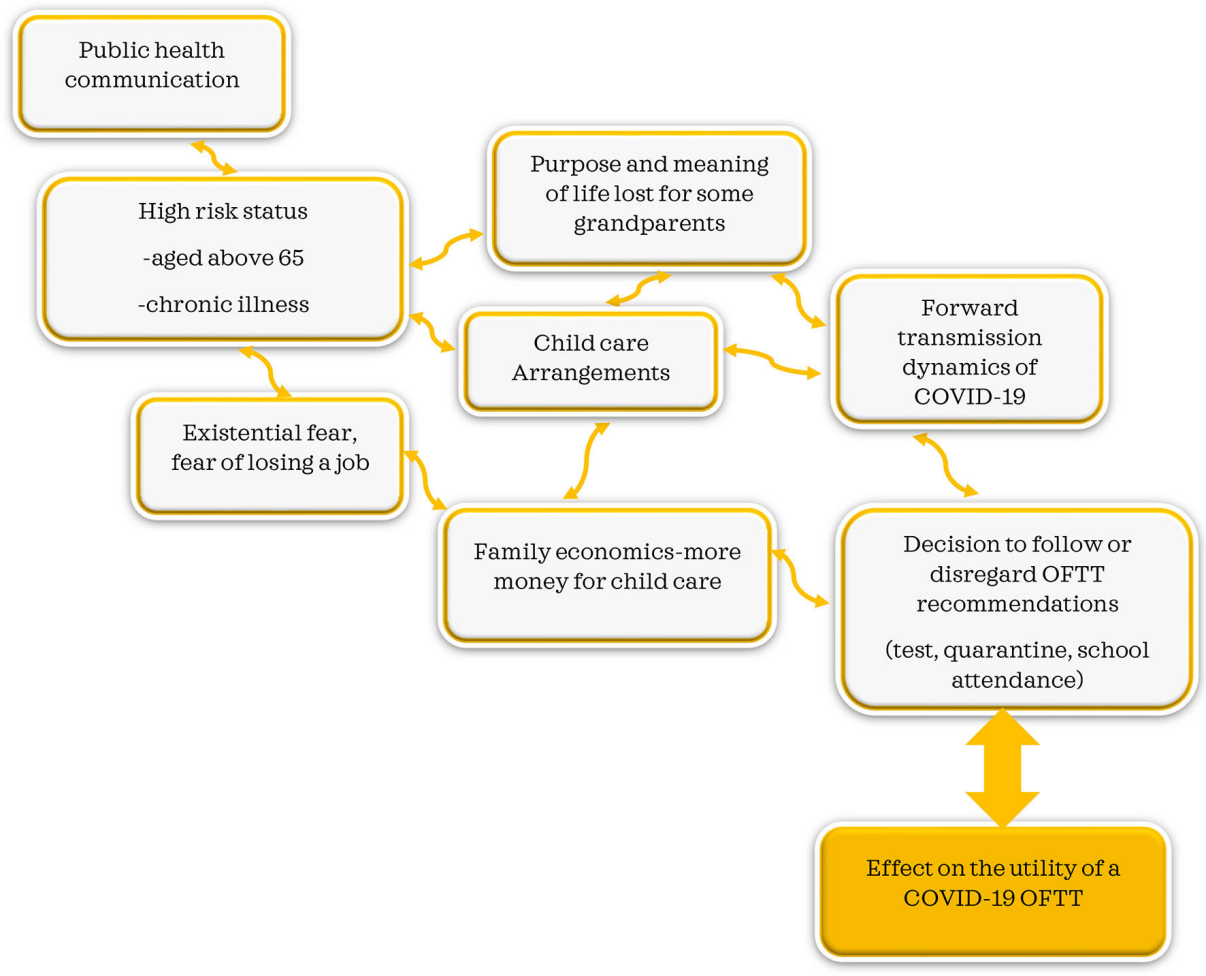
Public health communication interconnectedness and unintended consequences.

Our study sheds light on the complex decision-making process that confronts OFTT users, which goes beyond simple tool recommendations. The following themes emerged: (i) definition and expectations of high-risk persons, (ii) quarantine instructions and challenges, (iii) blurred responsibility between authorities and parents, (iv) a novel condition and the evolution of knowledge, (v) definition and implications of socioeconomic status, and (vi) new normal and societal divisions (Table 2, Figure 1).

### High-risk person definition and expectations

As everyone aged 65 years was regarded as being at high risk of suffering severe COVID-19 disease, a significant portion of the Swiss population was thought to require public health interventions (5, 22), and measures to protect this group were put in place. Our study revealed a perceived lack of information about what to do if you are a parent and at high risk. Living with a person who was considered high risk, a partner, or a child, generated significant uncertainties and challenges within families (5, 23).

Many Swiss families rely on grandparents who take care of their grandchildren on a regular basis (24). The high-risk assignation of those aged 65 years and above brought in an additional dynamic into many families. With instructions to protect grandparents, many families had to reorganize childcare, and it is needless to say that the childcare system in Switzerland is quite expensive (22). In line with our findings, many grandparents felt disenfranchised by their usefulness and their role in the family in taking care of grandchildren. For them, the experience was described as both isolating and disorienting, as many grandparents find purpose and meaning in caring for grandchildren (2, 24, 25). Significant social isolation-induced psychological effects have been reported in this group (11, 22, 24, 26). In concurrence with our findings, socioeconomic status, particularly finances, and influences perceived stress levels (7, 23, 27). With grandparents out of the childcare equation, childcare became an additional economic strain over and above the many stressors of life in general and the pandemic in particular (23, 27). With lack of grandparent support, additional childcare costs, and fear of losing one's job, testing and quarantine recommendations become challenging and difficult to follow, in concurrence with other findings (7, 27, 28). The effect on OFTT utility, testing, and forward transmission of the virus need to be considered and explored further (Figure 1).

### Socioeconomic status, quarantine instructions, and challenges

The quarantine experience was perceived very differently by the key informants in our study. Some described it as stressful or even enriching depending on the family environment, living space, and profession. The first quarantine experiences were described by many as adventurous, with more family time. However, some families experienced serial quarantines, disruptions in social life, and having to reorganize work and family life, which resulted in testing and quarantine fatigue (6). Homeschooling emerged as another major challenge that families faced. These factors described in this study were also reported to be important factors in the decision to test or quarantine and thus may have a direct influence on the course of the pandemic and the effect of public health measures. Socioeconomic status shaped everyday life during the pandemic (4–7, 27). Whether a family lived in a house with a garden or a small apartment in the city emerged as an important factor influencing decisions to follow or disregard public health recommendations, highlighting the link between living arrangements and socioeconomic status. Links between socioeconomic status, living space, homeschooling, working from home, and stress levels have also been reported elsewhere (4, 6, 7, 27). Access to outdoor spaces needs to be considered in pandemic settings. The very term, “socioeconomic status” was not understood by some key informants, while some felt uncomfortable divulging their status (privacy). Other ways of assessing this in research are needed.

### The evolution of knowledge and societal divisions

One problem of many parents was to assess how infectious their children were. Many decisions were made around this question, and the novel nature of the disease made it difficult for both parents and OFTT providers. Clear communication of this issue influenced many decisions from testing, sending a child to school or visiting grandparents, or allowing them to take care of their grandchildren. In line with our findings, failure to engage the community and inadequate health risk communication can render the best public health strategies ineffective (29). The lack of clear information about what to do, accompanied by fear of contracting or passing on the disease, brought about societal divisions, the young vs. the old, and intrafamilial and interfamilial conflict. In support of our findings, fear, a parallel pandemic, has been reported elsewhere (30).

### Blurred responsibility between authorities and parents

Switzerland is a country that places high value on individual responsibility and has decentralized decision-making across its 26 cantons, especially involving the healthcare sector (5). The pandemic put the notions of decentralized decision-making and individual responsibility to the test. Policies on school attendance, test criteria, school testing, or the wearing of masks varied across cantons (5, 28, 31). Attending school is compulsory in Switzerland. Parents can therefore not keep their children out of school. Some parents were left helpless by the feeling of being responsible for their children and sometimes by fear of contracting COVID-19 on the one hand, but on the other hand, being forced to send the children to school by law.

Blurred responsibilities between parents and authorities further caused significant uncertainties about how to proceed when a child fell sick (31). The novel, evolving virus, knowledge about the virus, changing guidelines, and sometimes divergent recommendations made by authorities and schools made the situation difficult for parents: Who do I follow (32)? The federal government, canton, or school authorities? In support of our findings, clarity on the roles and responsibilities of different authorities in pandemic settings is called for (31, 32). Should authorities override the parental responsibility to protect their own children, possibly by keeping them at home when they fear a novel infection with unknown health consequences? Some parents highlighted the fact that the high-risk status of teachers and childcare personnel is not known, which further complicates the matter. Should teachers and childcare personnel disclose their status to parents in pandemic settings (5, 31). Such questions need to be explored in the future.

### Interconnectedness-systems thinking

Our study revealed the interconnectedness between public health communications and other factors, ranging from high-risk status and expectations, socioeconomic status, and medical and practical decisions to decisions to test quarantine or send the kid to school. The decision to follow or disregard OFTT recommendations is influenced by these broader issues. The interconnectedness revealed in our study highlights the need for systems thinking in public health communication since a policy can have both intended and unintended effects (33) (Figure 1).

## Lessons learned

Public health communication in a pandemic setting emerged as both critical and challenging. In line with our findings, it is imperative to note that in crises, communication that focuses more on information provision than on practical behavior may lead to a public that knows what to, without understanding the action to take, leading to non-adherence behaviors (34). Many key informants that used the child-specific COVID-19 OFTT revealed that the decision to follow or disregard recommendations is complex and multifaceted. We learned the following lessons which could be of use in future COVID-19 telehealth interventions:

- We have learned that indeed more effective communication strategies are called for in times of public health crises. Communication of high-risk person groups and the use of high-risk person labels in OFTTs and public health, in general, ought to be accompanied by clear instructions and measures to protect all identified groups and thus to prevent stigma. What role can digital health play? Can technological mediation get this right? We recommended evaluative research on digital interventions to further explore this issue.
- Public health communication and recommendations issued during the COVID-19 pandemic were cited as having an effect on family constructs, roles, and practical and medical decision-making. These effects on individuals and families need to be considered at present and in future telehealth interventions (OFTT).
- Isolation and quarantine emerged as a challenge for specific population groups with limited living space and limited access to outdoor spaces such as gardens. This aspect needs to be considered in future pandemics.
- Perceived blurred responsibility lines between parents and school authorities, federal government, and cantons were cited as challenges in our study. The perceived blurred responsibility lines seem to affect individual and family decision-making. This calls for further discussions.
- Policies can have both intended and unintended consequences. Attempts to protect older people can have unintended consequences for the older people themselves and the family in general.
- The multiple factors presented above reportedly influenced individual and family decision-making, attitudes to test or not to test, and quarantine experiences. These findings demonstrate and highlight the need for systems thinking in public health communication.

### Strengths and limitations

Our tool was one of the first child-specific COVID-19 OFTTs set up in Switzerland. The insights gained in this study can help inform other telehealth departments setting up OFTTs for public health communication at present and in future pandemic settings on the one hand but also inform healthcare authorities in their efforts to further improve public health policy communication.

Our study findings have the limitation that they derive from a specific health setting in Switzerland, and our key informants are users of a specific health communication tool, our COVID-19 children OFTT. Transferability to other societies, pandemics, and settings might be limited.

## Conclusion

Considering the evolving virus, rapidly changing circumstances, and different interest groups, public health communication becomes both an art and science, even more so when using a new technological communication channel, an OFTT. Policies can have both intended and unintended consequences. Public health communication strategies need to be continuously evaluated to ensure that intended messages are understood by the public. Our study findings, therefore, highlight the need for systems thinking in public health communication (33, 35, 36), especially in the pediatric context as policies affect family and societal structures.

## Data availability statement

The original contributions presented in the study are included in the article/Supplementary material, further inquiries can be directed to the corresponding author/s.

## Ethics statement

Ethics review and approval/written informed consent was not required as per local legislation and institutional requirements.

## Author contributions

JM, AM, CS, KK, and TS were involved in the study design and data collection. JM and JR carried out the qualitative data analysis and wrote the first draft. JM, JR, AM, CS, NT, CA, KK, and TS contributed to further drafts. All authors contributed and approved the final draft.

## Funding

This study was partly cofinanced by the Federal Office of Public Health, Switzerland, and the Swiss National Science Foundation (196615). Emergency telemedicine at the University of Bern, Switzerland was supported by the Touring Club Switzerland through a foundational professorship to TS. The funder has no influence on the content of the manuscript or the decision to publish it.

## Conflict of interest

TS holds the endowed professorship for emergency telemedicine at the University of Bern, Switzerland. The funder, Touring Club Switzerland, has no influence on the research performed, the content of any manuscript, or any decision to publish. The remaining authors declare that the research was conducted in the absence of any commercial or financial relationships that could be construed as a potential conflict of interest.

## Publisher's note

All claims expressed in this article are solely those of the authors and do not necessarily represent those of their affiliated organizations, or those of the publisher, the editors and the reviewers. Any product that may be evaluated in this article, or claim that may be made by its manufacturer, is not guaranteed or endorsed by the publisher.

## References

[1] Molloy J Schatzmann T Schoeman B Tchervenkov C Hintermann B Axhausen KW. Observed impacts of the Covid-19 first wave on travel behaviour in Switzerland based on a large GPS panel. Transp Policy. (2021) 104:43–51. 10.1016/j.tranpol.2021.01.009 PMC9759204 36569490

[2] Pagani A Fritz L Hansmann R Kaufmann V Binder CR. How the first wave of COVID-19 in Switzerland affected residential preferences. Cities Health. (2021) 0:1–13. 10.1080/23748834.2021.1982231

[3] *Coronavirus: Bundesrat erklärt die* ≪*ausserordentliche Lage*≫ *und verschärft die Massnahmen*. Available from: https://www.admin.ch/gov/de/start/dokumentation/medienmitteilungen.msg-id-78454.html (accessed January 24, 2022).

[4] swissinfo.ch/sm. Study Highlights Life and Death Impact of Swiss Lockdown Timing. SWI swissinfo.ch. Verfügbar unter: https://www.swissinfo.ch/eng/sci-tech/swiss-lockdown-timing-saved-thousands–claims-study/45929698 (accessed January 24, 2022).

[5] Refle JE Voorpostel M. First Results of the Swiss Household Panel – Covid-19 Study. (2020). Available from: https://forscenter.ch/working-papers/first-results-of-the-swiss-household-panel-covid-19-study/ (accessed January 24, 2022).

[6] Lockdown Led to Stress and Mental Health Problems among Children Adolescents Parents and Young Adults. Universität Zürich. Available from: http://www.media.uzh.ch/en/Press-Releases/2021/Lockdown-Stress.html (accessed January 24, 2022).

[7] Mohler-Kuo M Dzemaili S Foster S Werlen L Walitza S. Stress and mental health among children/adolescents, their parents, and young adults during the First COVID-19 lockdown in Switzerland. Int J Environ Res Public Health. (2021) 18:4668. 10.3390/ijerph18094668 33925743 PMC8124779

[8] Omicron, Officially Dominant in Switzerland,. The Local Switzerland. Available from: https://www.thelocal.ch/20211228/omicron-officially-dominant-in-switzerland (accessed January 24, 2022).

[9] *Update on Omicron*. Available from: https://www.who.int/news/item/28-11-2021-update-on-omicron (accessed December 6, 2021).

[10] *EU, Countries Tighten Travel Restrictions As Omicron Variant Is Detected in Several Member States*,.SchengenVisaInfo.com Available from: https://www.schengenvisainfo.com/news/eu-countries-tighten-travel-restrictions-as-omicron-variant-is-detected-in-several-member-states/ (accessed December 6, 2021).

[11] Moser A von Wyl V Höglinger M. Health and social behaviour through pandemic phases in Switzerland: Regional time-trends of the COVID-19 Social Monitor panel study. PLoS ONE. (2021) 16:e0256253. 10.1371/journal.pone.0256253 34432842 PMC8386858

[12] *Media Releases*. Available from: https://www.bag.admin.ch/bag/en/home/das-bag/aktuell/medienmitteilungen.html?dyn_startDate=01.01.2021 (accessed May 9, 2022).

[13] Seeger MW Pechta LE Price SM Lubell KM Rose DA Sapru S. A conceptual model for evaluating emergency risk communication in public health. Health Secur. (2018) 16:193–203. 10.1089/hs.2018.0020 29927343

[14] Communication and SARS-CoV-2 – Swiss National COVID-19 Science Task Force. Available from: https://sciencetaskforce.ch/en/policy-brief/communication-and-sars-cov-2-2/. 9. May 2022

[15] Hautz WE Exadaktylos A Sauter TC. Online forward triage during the COVID-19 outbreak. Emerg Med J. (2021) 38:106–8. 10.1136/emermed-2020-209792 33310732 PMC7735070

[16] Hautz WE Exadaktylos AK Sauter TC. Web-based forward triage during the COVID-19 outbreak. EMJ. Available online at: https://emj.bmj.com/content/38/2/106 10.1136/emermed-2020-209792 PMC7735070 33310732

[17] www.coronabambini.ch. Development and Usage of an Online Decision Support Tool for Pediatric COVID-Testing in Switzerland. JMIR Preprints. Verfügbar unter. Available online at: https://preprints.jmir.org/preprint/37538 (accessed March 9, 2022).

[18] Baxter P Jack S. Qualitative case study methodology: study design and implementation for novice researchers. Qual Rep. (2008) 13:544–59. 10.46743/2160-3715/2008.1573

[19] Ivankova NV Creswell JW Stick SL. Using Mixed-Methods Sequential Explanatory Design: From Theory to Practice. (2006). Available from:https://pdfs.semanticscholar.org/363c/fe5efa01349c3685a023950ffa552ae824bf.pdf?_ga=2.201592529.1389452148.1584978635-451654905.1584978635 (accessed March 9, 2022).

[20] Michel J Mettler A Mueller M Hautz WE Sauter TC. A Utility framework for COVID-19 online forward triage tools. Int J Environ Res Public Health. (2022) 19:5184. 10.3390/ijerph19095184 35564576 PMC9105154

[21] Michel J Reid S Dörlemann A Tanner M. Why the misinformation, shame and guilt associated with coronavirus? J Glob Health Rep. (2020) 4:e2020089. 10.29392/001c.17364

[22] FOPH FO of PH. Coronavirus: People at Especially High Risk. Available from:https://www.bag.admin.ch/bag/en/home/krankheiten/ausbrueche-epidemien-pandemien/aktuelle-ausbrueche-epidemien/novel-cov/krankheit-symptome-behandlung-ursprung/besonders-gefaehrdete-menschen.html (accessed March 9, 2022).

[23] Grasso M Klicperová-Baker M Koos S Kosyakova Y Petrillo A Vlase I. The impact of the coronavirus crisis on European societies. What have we learnt and where do we go from here? – Introduction to the COVID volume. Eur Soc. (2021) 23:S2–32. 10.1080/14616696.2020.1869283

[24] *Grandparents Are Indispensable*. Swiss Life Group. Available from: https://www.swisslife.com/en/home/hub/grandparents-are-indispensable.html (accessed February 9, 2022).

[25] Hayslip B Knight R P Page K S Phillips C. Thematic dimensions of gr thematic dimensions of grandparent car ent caregiving: A F egiving: a focus gr ocus group approach. Contemp J Res Pract Policy. (2020) 6. Available online at: https://scholarworks.wmich.edu/grandfamilies/vol6/iss1/5/

[26] Govender I. COVID-19 with social distancing, isolation, quarantine and cooperation, collaboration, coordination of care but with disproportionate impacts. South Afr Fam Pract. (2020) 62:2. 10.4102/safp.v62i1.5204 32896137 PMC7577338

[27] Spinelli M Lionetti F Pastore M Fasolo M. Parents' stress and children's psychological problems in families facing the COVID-19 outbreak in Italy. Front Psychol. (2020) 11:1713. 10.2139/ssrn.3582790 32719646 PMC7350926

[28] Kuhn U Klaas HS Antal E Dasoki N Lebert F Lipps O. Who is most affected by the Corona crisis? An analysis of changes in stress and well-being in Switzerland. Eur Soc. (2021) 23:S942–56. 10.1080/14616696.2020.1839671

[29] Vaughan E Tinker T. Effective health risk communication about pandemic influenza for vulnerable populations. Am J Public Health. (2009) 99(Suppl. 2):S324–32. 10.2105/AJPH.2009.162537 19797744 PMC4504362

[30] Michel J Stuber R Müller M Mettler A Furrer H Ferrand RA. COVID-19 and HIV testing: different viruses but similar prejudices and psychosocial impacts. J Glob Health Rep. (2021) 5:e2021022. 10.29392/001c.21403

[31] Biller-Andorno N Zeltner T. Individual responsibility and community solidarity–the swiss health care system. N Engl J Med. (2015) 373:2193–7. 10.1056/NEJMp1508256 26630139

[32] Keystone-SDA/jdp. Study: Federalism a Challenge Not an Obstacle in Covid-19 Response. SWI swissinfo.ch. Verfügbar unter: Available from: https://www.swissinfo.ch/eng/business/study–federalism-a-challenge-not-an-obstacle-in-covid-19-response/47193762 (accessed February 9, 2022).

[33] Michel J Chimbindi N Mohlakoana N Orgill M Bärnighausen T Obrist B. How and why policy-practice gaps come about: a South African Universal Health Coverage context. J Glob Health Rep. (2019) 3:e2019069. 10.29392/joghr.3.e2019069

[34] Sellnow T Parker J Sellnow D Littlefield R Helsel E Getchell M. Improving biosecurity through instructional crisis communication: lessons learned from the PEDv outbreak. J Appl Commun. (2017) 101. 10.4148/1051-0834.1298

[35] Michel J Obrist B Bärnighausen T Tediosi F McIntryre D Evans D. What we need is health system transformation and not health system strengthening for universal health coverage to work: Perspectives from a National Health Insurance pilot site in South Africa. South Afr Fam Pract. (2020) 62:15. 10.4102/safp.v62i1.5079 32896142 PMC8377795

[36] Senge PM. The Fifth Discipline: The Art and Practice of the Learning Organization. London: Random House Business Books (1999). 424 S p.

